# Medical Cannabinoids for Cancer Cachexia: A Systematic Review and Meta-Analysis

**DOI:** 10.1155/2019/2864384

**Published:** 2019-06-23

**Authors:** Jing Wang, Yanling Wang, Mengting Tong, Hongming Pan, Da Li

**Affiliations:** ^1^Department of Medical Oncology, Sir Run Run Shaw Hospital, Zhejiang University School of Medicine, Hangzhou, Zhejiang, China; ^2^Second Department of Oncology, The Fourth Affiliated Hospital of Xinjiang Medical University, Urumqi, Xinjiang 830001, China

## Abstract

**Objectives:**

Cancer cachexia (CCA) is an intractable and ineffective metabolic syndrome that attacks 50–80% of cancer patients. It reduces patient's life quality, affects the efficacy of treatment, and then increases their mortality; however, there are no established therapeutic strategies for CCA in the world. In this study, we assess the positive and negative effects of cannabinoid in the treatment of CCA.

**Methods:**

We searched the Cochrane Central Register of Controlled Trials (CENTRAL) in The Cochrane Library, MEDLINE, EMBASE, Web of Science, and PubMed up to December 2017.

**Results:**

Of the 256 screened studies, three studies with a total of 592 participants were included. Compared with placebo, cannabinoid increased the appetite (MD 0.27, 95% CI -0.51 to 1.04; n= 3) but failed to improve the overall quality of life (QOL; MD -12.39, 95% CI [-24.21 to -0.57; n = 2), and a total of 441 patients had 607 adverse events (AEs; 496 in the cannabinoid group and 111 in the placebo group).

**Conclusions:**

Our analysis showed cannabinoid is effective in increasing appetite in cancer patients. However, it declines the quality of life, which may be due to the side effects of cannabinoid.

## 1. Introduction

Cancer cachexia (CCA) is a multifactorial syndrome, which is the result from interaction between the host and tumors, characterized by weight loss, anorexia, and anemia [1]. CAAs affected approximately 50%–80% of the cancer patients [2] and represents the direct cause of at least 25% of cancer deaths [3], due to the long-term lack of nutritional intake, digestion, and absorption dysfunction [4]. Subsequently, malnutrition could affect the effectiveness of drug therapy by increasing chemotherapy toxicity and decreasing the positive influence on tumor treatment. Nevertheless, increasing in food intake does not prevent this negative outcome, and unfortunately, there is no effective therapy for cachexia so far.

Successful tumor treatment requires the implementation of a comprehensive multidisciplinary approach. Radiotherapy, chemotherapy, surgical resection, and other treatment programs can aggravate the weakness of cancer patients. Therefore, anti-cachexia treatment must address the following important therapeutic aspects: (1) correction of the cachexia status; (2) control of the symptoms; (3) amelioration of bodily functions; and (4) improvement of the quality of life. In general, anti-cachexia treatment should not only improve the quality of life (QOL) but also promote the effectiveness of cancer treatment.

So far, there are no standard guidelines for cachexia treatment. However, many drugs are developed for cachexia therapy, including glucocorticoids, cytokine receptors, progestogens, non-steroidal anti-inflammatory drugs, beta-adrenoceptor agonists, and cannabinoids. Of them, progestogens, such as medroxyprogesterone and megestrol, are the only drugs approved by FDA for cancer-related cachexia [5]. Although their anti-cachectic effect is associated with the improvement of anorexia and body weight as well as of quality-of-life parameters, they can only increase adipose tissue and have not been confirmed to augment lean body mass [6]. Besides, in the United States and the United Kingdom, THC is considered to be effective in the treatment of cachexia [7]. Previous studies showed that cannabinoid had considerable potential to improve the appetite, body weight, body fat level, caloric intake, mood, quality of life and reduced pain, nausea, and vomiting in patients with multiple sclerosis [8], HIV [9], anorexia nervosa [10], obesity [11], and type 2 diabetes [12]. Furthermore, this bioactive substance attracted intensive research interest for application in cardiac cachexia treatment [13].

The mechanism of cannabinoid activity is related to G-protein coupled with cannabinoid 1 and 2 receptors (CB1/2). CB1 are mostly found in the brain and CB2 receptors are found on all cell types. Above on, it can exert a moderating effect on obesity, metabolism, and pain by activating pathways that are cannabinoid receptors scattered all over the body (Kaminska et al., 2015) and then may play important roles in weight gain, increased appetite, decreased nausea, improvement in mood, relief of pain, and so on.

In this study, we searched the Cochrane Central Register of Controlled Trials, MEDLINE, EMBASE, Web of Science, and PubMed up to December 2017. Of the 256 screened studies, 3 studies with a total of 592 participants were included which match our criteria. We assessed whether cannabinoid is effective for CCA by investigating appetite and quality of life. We think our analysis will explore whether cannabinoid is effective for CCA and provide a basis for guiding clinical drug use.

## 2. Methods

### 2.1. Study Selection Criteria

Inclusion criteria were based on the PICOS acronym (participant, intervention, comparison, outcomes of interest, and study design). As a participant population (P), all cancer patients were treated with cannabinoids. Intervention (I) and comparison (C) denoted the studies that had comparatively investigated the effects of cannabinoids versus placebo. As an outcome of interest (O), we accessed the following outcomes: appetite, overall quality of life (QOL), body weight, and therapy-related adverse events (AEs). Regarding the study design (S), only double-blind, randomized clinical trials (RCTs) were considered. The exclusion criteria were as follows: healthy volunteers; subjects under 18 years of age; patients with either normal nutrition or obesity; receiving enteral or parenteral nutrition; brain, breast, ovarian, or endometrial cancer; a study that reported at least one of the above outcome measures; animal studies; absence of a placebo treatment as a control; and non-RCT.

### 2.2. Search Methods for Identification of Studies

**Figure 1:**
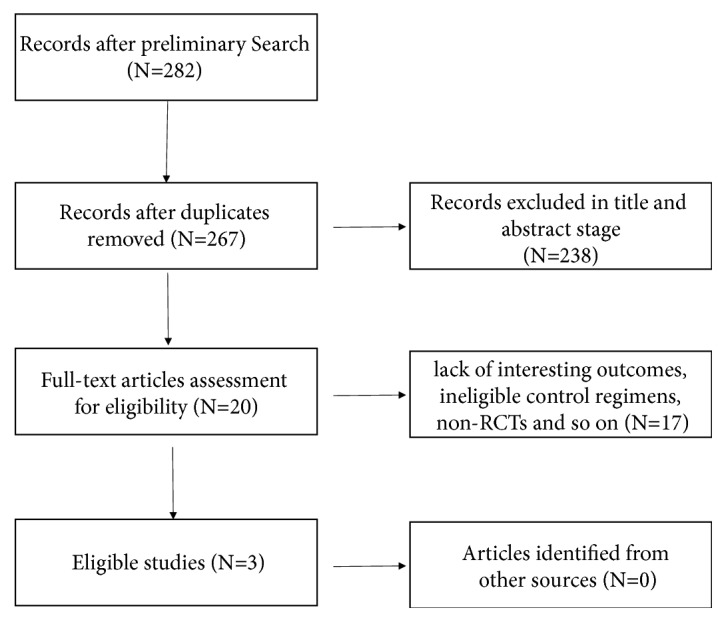
A flowchart of study selection.

We used “Cachexia”, “emaciation”, “wasting syndrome”, “Cannabis”, “cannabinoid”, “body weight”, “muscular atrophy”, and “Dronabinol” as the keywords to search the RCTs in Cochrane Central Register of Controlled Trials (CENTRAL), MEDLINE, Web of Science, EMBASE, PubMed, as well as China Academic Journals full-text database, also known as CNKI (updated until December 2017). The scope of the search was exceedingly broad, and detailed process is shown in Figure 1.

### 2.3. Data Extraction

Two independent investigators extracted relevant information on participant characteristics, interventional protocol, follow-up, outcome data, and risk assessment of bias. Any disagreement was solved by either consensus or consultation with a third author (LD), followed by discussion. The inclusion/exclusion criteria and main characteristics of the three included studies are presented in Table 1.

### 2.4. Quality Assessment

Risk of bias was assessed using the criteria list advised by the quality assessment of diagnostic accuracy studies, which consists of 4 parts: patient selection, index test, reference standard, and flow of patients through the study and timing of the index tests and reference standard (flow and timing). Two researchers independently screened the literature, extracted data, and performed a cross-check. In cases of a disagreement, the discussion was resolved or referred to the third researcher.

### 2.5. Assessment of the Risk of Bias in the Included Studies

The risk of bias of the RCTs, including detection bias, selection bias, reporting bias, performance bias, attrition bias, and other potential bias, was evaluated by Review Manager 5.3 software (Cochrane Collaboration, Copenhagen, Denmark) and STATA software (version 12.0, StataCorp, TX, USA).

### 2.6. Patient and Public Involvement

Our current study is a systematic review based on published data, so the patient and the public are not involved in the study design, conduct, data analysis, and result dissemination.

## 3. Results

### 3.1. Participants

At the initial search stage, 273 potentially relevant studies were retrieved based on the above search strategy (Figure 1). After screening the title and abstract, 19 studies were selected for the full-text assessment, of which 16 trials were excluded due to several reasons, such as ineligible control regimens, and non-RCTs. All selection procedures were performed independently by two investigators. Finally, our study included a total of 592 patients from three studies [14–16] of which 466 patients were randomized to the cannabinoids group (n = 337) and the control group (n = 129). All included RCTs compared the efficacy of cannabinoids with a corresponding placebo and patients with cancer.

### 3.2. Risk of Bias in the Included Studies

**Figure 2:**
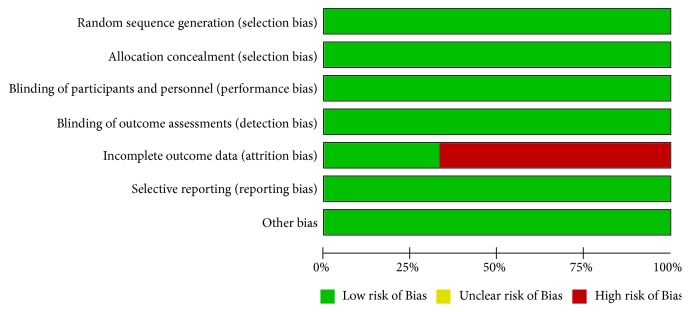
Risk of bias graph: review authors' judgements about each risk of bias item presented as percentages across all included studies.

First, we assessed the risk of bias according to the Cochrane reviewers' handbook. We found the attrition bias in two of the studies was over 20%; however, this was attributed to patient withdrawal without intention to treat analysis and per protocol simultaneously [15, 16] as shown in Figure 2. Nevertheless, all of the trials appeared to be free of the other bias. Hence, the statistical quality of all studies was reliable.

### 3.3. Cannabinoids Treatment Increases Appetite

**Figure 3:**
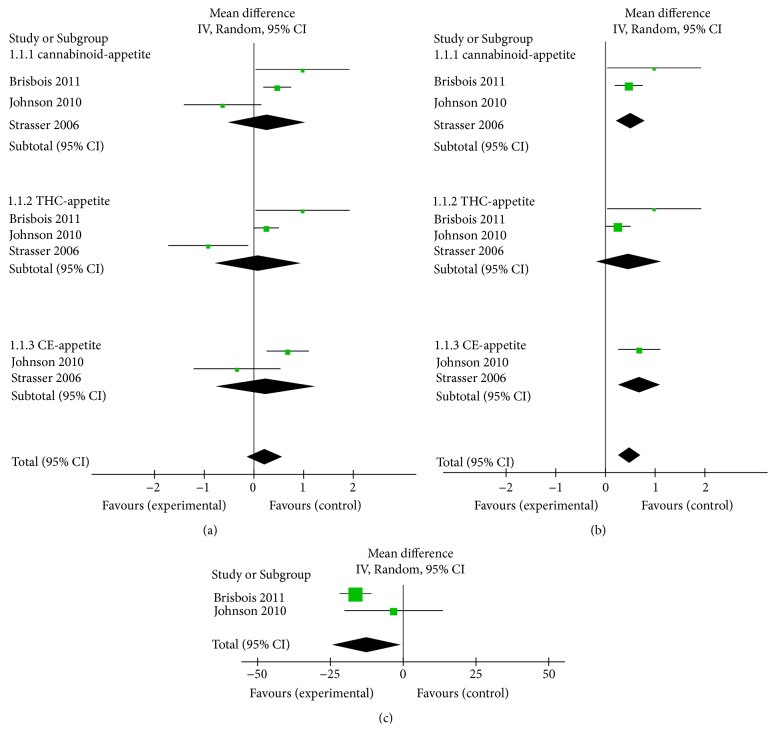
(a) Comparison: cannabinoids versus placebo, outcome: appetite (MD 0.27, 95% CI -0.51 to 1.04; n = 3). (b) Comparison: cannabinoids versus placebo, outcome: appetite after sensitivity analysis (MD 0.52, 95% CI 0.23 to 0.81; n = 2). (c) Comparison: cannabinoids versus placebo, outcome: overall QOL (MD -12.39, 95% CI [-24.21 to -0.57; n = 2).

It has been shown that cannabinoid has a potential role in increasing appetite. In this study, the data of appetite were available from 330 participants. Our analysis indicated that cannabinoids have the tendency to associate with increasing appetite (MD 0.27, 95% CI: [-0.51 to 1.04], Z = 0.68, P = 0.50, chi2 = 8.39, P for heterogeneity = 0.02, I2 = 76%), while in subgroup analysis, appetite in THC (MD 0.09, 95% CI -0.77 to 0.95; n = 3) and CE (MD 0.24, 95% CI -0.74 to 1.23; n = 2) group was similarly increased with cannabinoid treatment (Figures 3(a) and S1). Sensitivity analyses (Figures 3(b) and S2) revealed that the pooled estimate effect and heterogeneity were significantly improved after the exclusion of the study of Strasser [15] (MD 0.52, 95% CI: [0.23 to 0.81], Z = 3.51, P = 0.0005, chi2 = 1.03, P for heterogeneity = 0.31, I2 = 3%). Hence, in the subgroup of THC and CE, we can draw a clear conclusion that treatment of THC and CE can increase appetite in cancer patients, and there is no significant difference between these groups.

### 3.4. Cannabinoids with the Patient Overall Quality of Life

We next analyzed the assessment results of overall QOL of cancer patients. In our meta-analysis, two studies described relevant statistical data on the overall QOL of cancer patients. Interestingly, there is no improvement in the quality of life of the patients in the cannabinoids group compared with the placebo group. Surprisingly, we found that the trend was even the opposite (MD -12.39, 95% CI: [-24.21 to -0.57], Z = 2.06, P = 0.04, chi2 = 2.09, P for heterogeneity = 0.15, I2 = 52%, n = 2, Figures 3(c) and S3).

### 3.5. Publication Bias

**Figure 4:**
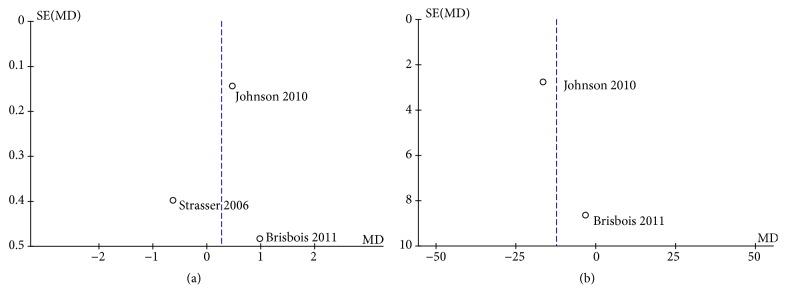
(a) Funnel plot of comparison: 1 cannabinoids versus placebo, outcome: appetite. (b) Funnel plot of comparison: 1 cannabinoids versus placebo, outcome: Overall QOL.

The funnel plot of HRs of the appetite was symmetric (Figure 4(a)), and the publication bias assessed by Egger's or Begg's test was also not significant (P = 0.789, P = 1, respectively). Symmetry in the funnel plot was also observed for the overall quality of life (Figure 4(b)), and the publication bias assessed by Begg's test was also not significant (P = 1). However, the number of studies for the QOL is 2, which is too small to make Egger's test.

## 4. Discussion

CCA can occur at any stages of malignancy. Weight loss is one of the red alerts for cachexia patients. Although our research on the biological characteristics and mechanisms of CCA has made some achievements in the past decades, there are still many unknowns that need to be determined. Although there are lots of evidence that cannabinoids are useful for the treatment of various medical conditions, the meta-analysis for cancer and cannabinoid is not well studied. To systematically analyze the role of cannabinoid in the therapy of CCA, we finally selected 3 studies into our meta-analysis, and 2 indicators, namely, appetite, and quality of life, used to evaluate the system. We showed that cannabinoid has the potency of improving symptoms, increasing the weight and improving the QOL of the patients with CCA.

The meta counted the appetite and overall QOL data results from the included 466 patients. Under the random-effects model, the results showed that MD was 0.27, P = 0.50, I2 = 76%. There was high evidence for heterogeneity across studies (I2 = 76%, p = 0.02), mostly accounted for the lower rate of increased appetite (58%) reported by Florian Strasser in CE group than placebo group (69%). Then we removed this study from the meta-analysis, followed that the I2 statistic reduced to 3%, OR changed to 0.52 with 95% CI 0.23-0.81, p = 0.0005, close to significantly different. The data suggest that cannabinoid can significantly increase the patient's appetite, but more clinical trials with large cases are needed to confirm it. However, as for overall QOL of cancer patients, the meta even support the theory that cannabinoid is counterproductive to the overall QOL of cachexia patients (MD -12.39, P = 0.04, I2 = 52%). The results show that the clinical application of cannabinoid in the treatment of patients with CCA may be at the expense of declining QOL.

The safety issue is still the bottom line in ascertaining the efficacy and practicality of a drug. Treatment-related AEs in the present meta-analysis included nausea, fatigue, pain, anemia, dizziness, dyspnea, diarrhea, obstipation, somnolence, raised *γ*-GT, hypercalcemia, hypotension, and so on. A total of 441 patients had 607 AEs (496 in the cannabinoids and 111 in the placebo groups) in the three studies. However, precise, detailed information could not be obtained as we received no reply after attempting to contact the authors. Besides, a multi-institutional study in AIDs patients found that dronabinol use was safe and well tolerated [17]. Though we cannot simply speculate that there was a higher risk of AEs in the cannabinoids group, it seems that cannabinoids are not absolutely safe for CCA patients. Of course, our conclusions may require more detailed data to increase the argument strength.

In this study, all included studies were RCTs, with cancer cachexia patients; hence the quality of this article was relatively high. However, there are also some limitations to this meta-analysis. Firstly, the results of three trials may be subject to the heterogeneity of study participants, including ethnicity, gender distribution, differences in treatment protocols, and patient sample sizes, leading to difficulties in exploring potential sources of heterogeneity and bias. Secondly, the results of the therapeutic effects of cannabinoid in CCA treatment are different, which may be related to the different dosage of cannabinoid. The dosage of cannabinoid is also a condition that determines its impact and treatment outcome. Of note, the appetite-increasing effect of dronabinol was established to occur at a lower dose than that needed to induce its antiemetic effect [13]. Dose-dependent effects on appetite, nausea, mood, perception, memory, pain, exercise, breathing, and intraocular pressure should arise in our attention. In the future, more clinical studies and adequate follow-up periods are required to authenticate the importance of the potential of cannabinoids for the prevention and management of CCA.

## Figures and Tables

**Table 1:** Research summaries comparing the effects of cannabinoids and placebo.

Study ID	Study method	Human subjects	Interventional group	Control group	Follow-up (d)	Outcomes
Brisbois 2011	parallel-group RCTs	advanced cancer patients	THC (2.5 mg, n = 24)	Placebo (n = 22)	19	Appetite, AEs, QOL
Johnson 2010	parallel-group RCTs	advanced cancer patients	THC:CBD extract (2.7 mg THC and 2.5 mg CBD, n = 60), THC extract (2.7 mg THC, n = 58)	Placebo (n = 59)	14	Appetite, AEs, QOL
Strasser 2006	parallel-group RCTs	advanced cancer patients	CE (standardized for 2.5 mg THC and 1 mg cannabidiol, n = 95) or THC (2.5 mg, n = 100)	Placebo (n = 48)	42	Body Weight, Appetite, AEs, QOL

## Data Availability

The dataset can be requested by sending an email to the corresponding author.

## Supplementary Materials

Supplementary Materials Figure S1: association of subgroup by different drugs on appetite on study. Figure S2: sensitivity analyses of subgroup by different drugs on appetite on study. Figure S3: meta-analysis of subgroup by different drugs on overall quality of life on study.
