# Supplementary material for: Medical Cannabinoids for Cancer Cachexia: A Systematic Review and Meta-Analysis
Source: Biomed Res Int. 2019 Jun 23;2019:2864384. doi: 10.1155/2019/2864384 (PMC6612387; doi:10.1155/2019/2864384)
Supplement: Supplementary Materials — Figure S1: association of subgroup by different drugs on appetite on study. Figure S2: sensitivity analyses of subgroup by different drugs on appetite on study. Figure S3: meta-analysis of subgroup by different drugs on overall quality of life on study. [file 2864384.f1.pdf]

Figure S1

Study or Subgroup	Experimental Mean	Experimental SD	Experimental Total	Control Mean	Control SD	Control Total	Weight	Mean Difference IV, Random, 95% CI
**1.1.1 cannabinoid-appetite**								
Brisbois 2011	6.07	1.13	11	5.09	1.08	10	8.4%	0.98 [0.03, 1.93]
Johnson 2010	4.86	1.2	118	4.39	0.7	59	18.5%	0.47 [0.19, 0.75]
Strasser 2006	3.55	2.2	183	4.18	2.38	43	10.4%	-0.63 [-1.41, 0.15]
**Subtotal (95% CI)**			**312**			**112**	**37.3%**	**0.27 [-0.51, 1.04]**
Heterogeneity: Tau² = 0.35; Chi² = 8.39, df = 2 (P = 0.02); I² = 76%								
Test for overall effect: Z = 0.68 (P = 0.50)								
**1.1.2 THC-appetite**								
Brisbois 2011	6.07	1.13	11	5.09	1.08	10	8.4%	0.98 [0.03, 1.93]
Johnson 2010	4.64	0.7	58	4.39	0.7	59	18.9%	0.25 [-0.00, 0.50]
Strasser 2006	3.26	1.85	92	4.18	2.38	43	10.1%	-0.92 [-1.73, -0.11]
**Subtotal (95% CI)**			**161**			**112**	**37.4%**	**0.09 [-0.77, 0.95]**
Heterogeneity: Tau² = 0.45; Chi² = 10.19, df = 2 (P = 0.006); I² = 80%								
Test for overall effect: Z = 0.21 (P = 0.83)								
**1.1.3 CE-appetite**								
Johnson 2010	5.07	1.52	60	4.39	0.7	59	16.1%	0.68 [0.26, 1.10]
Strasser 2006	3.84	2.47	91	4.18	2.38	43	9.2%	-0.34 [-1.21, 0.53]
**Subtotal (95% CI)**			**151**			**102**	**25.3%**	**0.24 [-0.74, 1.23]**
Heterogeneity: Tau² = 0.40; Chi² = 4.24, df = 1 (P = 0.04); I² = 76%								
Test for overall effect: Z = 0.48 (P = 0.63)								
**Total (95% CI)**			**624**			**326**	**100.0%**	**0.22 [-0.14, 0.57]**
Heterogeneity: Tau² = 0.16; Chi² = 24.90, df = 7 (P = 0.0008); I² = 72%								
Test for overall effect: Z = 1.21 (P = 0.23)								
Test for subgroup differences: Chi² = 0.10, df = 2 (P = 0.95), I² = 0%								

Figure S2

Study or Subgroup	Experimental Mean	Experimental SD	Experimental Total	Control Mean	Control SD	Control Total	Weight	Mean Difference IV, Random, 95% CI
**1.1.1 cannabinoid-appetite**								
Brisbois 2011	6.07	1.13	11	5.09	1.08	10	5.4%	0.98 [0.03, 1.93]
Johnson 2010	4.86	1.2	118	4.39	0.7	59	32.9%	0.47 [0.19, 0.75]
Strasser 2006	3.55	2.2	183	4.18	2.38	43		Not estimable
**Subtotal (95% CI)**			**129**			**69**	**38.3%**	**0.52 [0.23, 0.81]**
Heterogeneity: Tau² = 0.00; Chi² = 1.03, df = 1 (P = 0.31); I² = 3%								
Test for overall effect: Z = 3.51 (P = 0.0005)								
**1.1.2 THC-appetite**								
Brisbois 2011	6.07	1.13	11	5.09	1.08	10	5.4%	0.98 [0.03, 1.93]
Johnson 2010	4.64	0.7	58	4.39	0.7	59	36.2%	0.25 [-0.00, 0.50]
Strasser 2006	3.26	1.85	92	4.18	2.38	43	0.0%	-0.92 [-1.73, -0.11]
**Subtotal (95% CI)**			**69**			**69**	**41.6%**	**0.47 [-0.19, 1.12]**
Heterogeneity: Tau² = 0.14; Chi² = 2.14, df = 1 (P = 0.14); I² = 53%								
Test for overall effect: Z = 1.40 (P = 0.16)								
**1.1.3 CE-appetite**								
Johnson 2010	5.07	1.52	60	4.39	0.7	59	20.1%	0.68 [0.26, 1.10]
Strasser 2006	3.84	2.47	91	4.18	2.38	43		Not estimable
**Subtotal (95% CI)**			**60**			**59**	**20.1%**	**0.68 [0.26, 1.10]**
Heterogeneity: Not applicable								
Test for overall effect: Z = 3.14 (P = 0.002)								
**Total (95% CI)**			**258**			**197**	**100.0%**	**0.49 [0.26, 0.72]**
Heterogeneity: Tau² = 0.02; Chi² = 5.94, df = 4 (P = 0.20); I² = 33%								
Test for overall effect: Z = 4.17 (P < 0.0001)								
Test for subgroup differences: Chi² = 0.47, df = 2 (P = 0.79), I² = 0%								

Figure S3

Study or Subgroup	Experimental Mean	Experimental SD	Experimental Total	Control Mean	Control SD	Control Total	Weight	Mean Difference IV, Random, 95% CI
Johnson 2010	35.97	15.85	93	52.36	15.95	51	69.4%	-16.39 [-21.83, -10.95]
Brisbois 2011	98.5	20.2	11	101.8	19.3	10	30.6%	-3.30 [-20.20, 13.60]
**Total (95% CI)**			**104**			**61**	**100.0%**	**-12.39 [-24.21, -0.57]**

Heterogeneity: $Tau^2 = 44.66$; $Chi^2 = 2.09$, df = 1 (P = 0.15); $I^2 = 52\%$
Test for overall effect: Z = 2.06 (P = 0.04)
